# *Plasmodium falciparum* Adhesion on Human Brain Microvascular Endothelial Cells Involves Transmigration-Like Cup Formation and Induces Opening of Intercellular Junctions

**DOI:** 10.1371/journal.ppat.1001021

**Published:** 2010-07-29

**Authors:** Ronan Jambou, Valery Combes, Marie-Jose Jambou, Babeth B. Weksler, Pierre-Olivier Couraud, Georges E. Grau

**Affiliations:** 1 Vascular Immunology Unit, Department of Pathology and Bosch Institute, Sydney Medical School, The University of Sydney, New South Wales, Australia; 2 Department of Parasitology Mycology, Institut Pasteur, Paris, France; 3 Institut Cochin, CNRS UMR 8104, INSERM U567, Université Paris Descartes, Paris, France; 4 Cornell University Medical College, New York, New York, United States of America; Case Western Reserve University, United States of America

## Abstract

Cerebral malaria, a major cause of death during malaria infection, is characterised by the sequestration of infected red blood cells (IRBC) in brain microvessels. Most of the molecules implicated in the adhesion of IRBC on endothelial cells (EC) are already described; however, the structure of the IRBC/EC junction and the impact of this adhesion on the EC are poorly understood. We analysed this interaction using human brain microvascular EC monolayers co-cultured with IRBC. Our study demonstrates the transfer of material from the IRBC to the brain EC plasma membrane in a trogocytosis-like process, followed by a TNF-enhanced IRBC engulfing process. Upon IRBC/EC binding, parasite antigens are transferred to early endosomes in the EC, in a cytoskeleton-dependent process. This is associated with the opening of the intercellular junctions. The transfer of IRBC antigens can thus transform EC into a target for the immune response and contribute to the profound EC alterations, including peri-vascular oedema, associated with cerebral malaria.

## Author Summary

Cerebral malaria, a major cause of death during malaria infection, is characterised by the sequestration of infected red blood cells (IRBC) in brain microvessels. This study describes the interactions between plasmodium infected red blood cell and human brain endothelial cells. It highlights the activation of a trogocytosis-like mechanism followed by an engulfment of the infected red blood cells by endothelial cells (EC). This transfer concerns up to 20% of the IRBC cocultured with EC. This means that the parasite infected erythrocyte can mimic the leukocytes interaction with endothelial cells. This process is associated with i) a transfer of malaria antigens to the EC which can inappropriately activate the immune system and ii) an opening of the intercellular junctions, which can trigger blood-brain-barrier leakage during cerebral malaria. This transfer of IRBC antigens can thus transform EC into a target for the immune response and contribute to cerebral malaria pathogenesis.

## Introduction

Each year 3.2 billion people worldwide are exposed to the threat of malaria, resulting in around 2 million deaths [1]. Even with the best antiparasitic treatments, patients with cerebral malaria (CM) have no significant improvement in their prognosis, with an average fatality rate of 30 to 50% [1]. This outcome is due to a neurovascular pathology characterised by the accumulation of both infected red blood cells (IRBC) and host cells (leucocytes and platelets) in deep brain microvessels, leading to microcirculation impairment [2].

Leakage of the blood-brain-barrier and local arrest of leucocytes is associated with cytoadhesion of IRBC and microcirculation impairment, increased blood volume due to sequestration, and increased blood flow resulting from seizures and anaemia [2]. Cytokines and parasite toxins have also been shown to cause direct damage to the blood-brain barrier [3], [4]. On the whole, severe increased intracranial pressure [2] and brain oedema [5] are associated with poor outcome for patients with CM.

Of note however, the sequestration of IRBC in deep vessels is a normal step in the life cycle of the *Plasmodium* parasite and does not always trigger severe disease. Most of the molecules implicated in the adhesion of IRBC on EC have previously been described. They involve a series of endothelial molecules and parasite membrane proteins such as PfEMP1 and RIFINS [6]. This cytoadhesion helps the parasite avoid splenic clearance and favours its development in a low oxygen pressure microenvironment. Specific localisation of the parasites in the brain seems to be a complex feature involving both expression of human adhesion molecule isoforms and parasitic *var* proteins polymorphism [7]. However, the fine mechanisms of microvascular endothelial cell alterations are not yet fully understood.

Cellular interactions in microvessels have mostly been described in the context of transmigration of cells through the endothelial layer. Such transmigration occurs through either openings between adjacent endothelial cells or through a single endothelial cell, mainly when leukocytes migrate to tissues or during the diffusion of metastatic cells. This is a stepwise process involving rolling, adhesion, firm adhesion, and finally diapedesis. The formation of a docking structure or “transmigratory cup” was recently described and involves several endothelial adhesion and signalling molecules [8]–[10]. Human lymphocytes use another process and palpate the surface of EC with podosomes before forming transcellular pores through the endothelium [11]. Yet another mechanism of cell-cell interaction, named trogocytosis, was more recently described in mice and human immune cells, but not in EC. Trogocytosis involves the transfer of membrane compounds during short term cell interactions [12]–[13], and is defined as the uptake of membrane fragments and associated molecules from one cell to another. Moreover cell interaction by any of the above mechanisms can activate EC. Therefore, it is essential to understand the binding mechanism and the repercussions of the IRBC adhesion on EC in order to develop new preventive or therapeutic interventions for the treatment of cerebral malaria treatment.

The present study demonstrates, that IRBC undergo close association with EC in a manner reminiscent of both trogocytosis and transmigration. In this in vitro system, adhesion and transfer of material involved 10 to 20% of the IRBC in contact with the EC. This process involves engagement of ICAM-1, or other EC adhesion molecules, in the binding of IRBC. It triggers transfer of membrane material and malaria antigens from the IRBC to the brain EC in a trogocytosis-like manner and in a second step the development of a transmigration cup-like structure, which results in EC activation and opening of the intercellular junctions.

## Results

### Adhesion of IRBC to human brain EC is associated with a rapid transfer of IRBC membrane material

**Figure 1:**
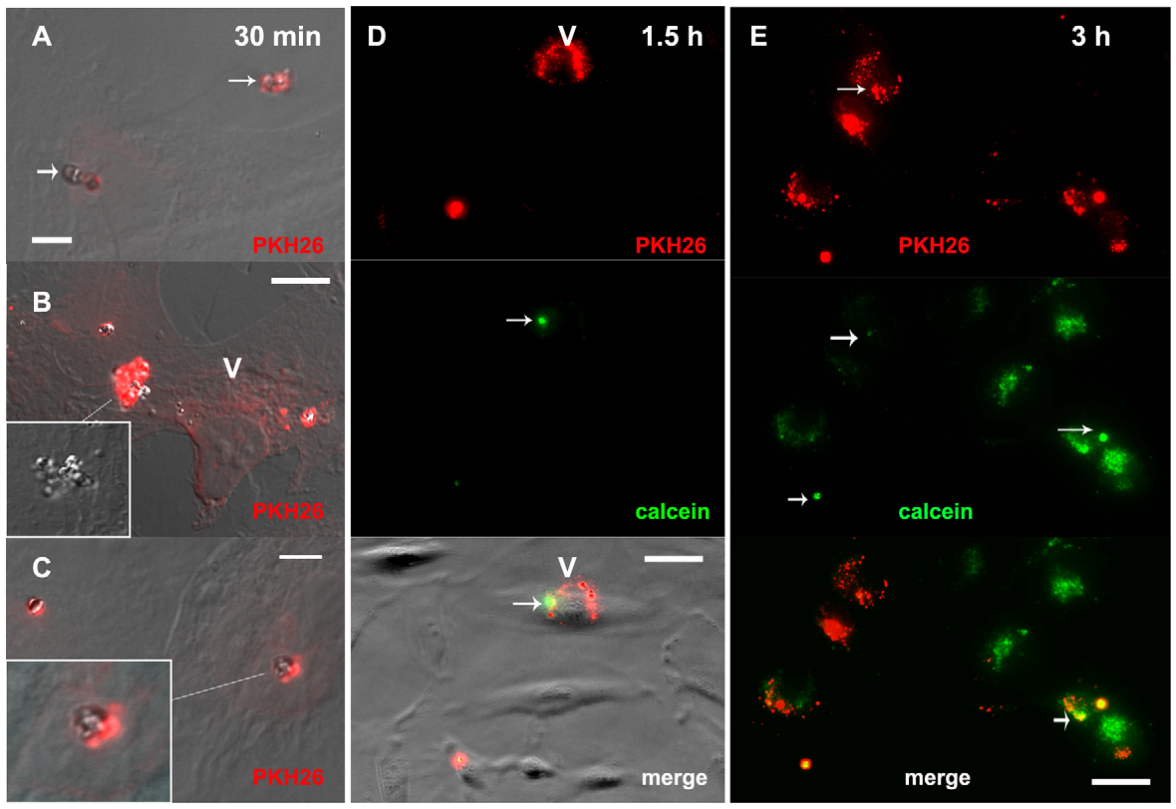
Transfer of material from IRBC to HBEC. Aspects of the HBEC-D3 monolayer after incubation with IRBC (3Ci strain) for 30 min (A–C), 1.5 h (D) or 3 h (E). Prior to co-culture, plasma membranes of the IRBC were labelled with PKH-26 (red) and cytoplasm with calcein (green). IRBC were added at a ratio of 20:1 i.e. 2 10^6^ IRBC per well of 24 wells plate. HBEC monolayers were examined by fluorescence microscopy at magnification 600 after fixation labelling and mounting as described. For A-B-C-D fluorescence and DIC pictures are merge to show HBED-D3 (V) and IRBC (arrow), except for insert 1B where only DIC is shown to visualize IRBC packed in an engulfing cup. Bar: 10 µm (A–C), 20 µM (D–F); (V) HBED-D3, (arrow) IRBC. Panels A to C show an early trogocytosis- like transfer of dye from the IRBC membrane onto the HBEC surface which appears as diffusion of red around the IRBC (arrows and insert 1C); in D, after 1.5 h of co-culture PKH26 is transferred in the HBEC (V) far from the IRBC still identified within the HBEC as green elements; a remaining normal red cell (PHK-26 labelled but poorly calcein-labelled) can be seen in the lower corner (arrow). E: show diffusion of the calcein in the HBEC after 3 h of culture with some remaining IRBC cytoplasm identified as green dots (arrow).

**Figure 2:**
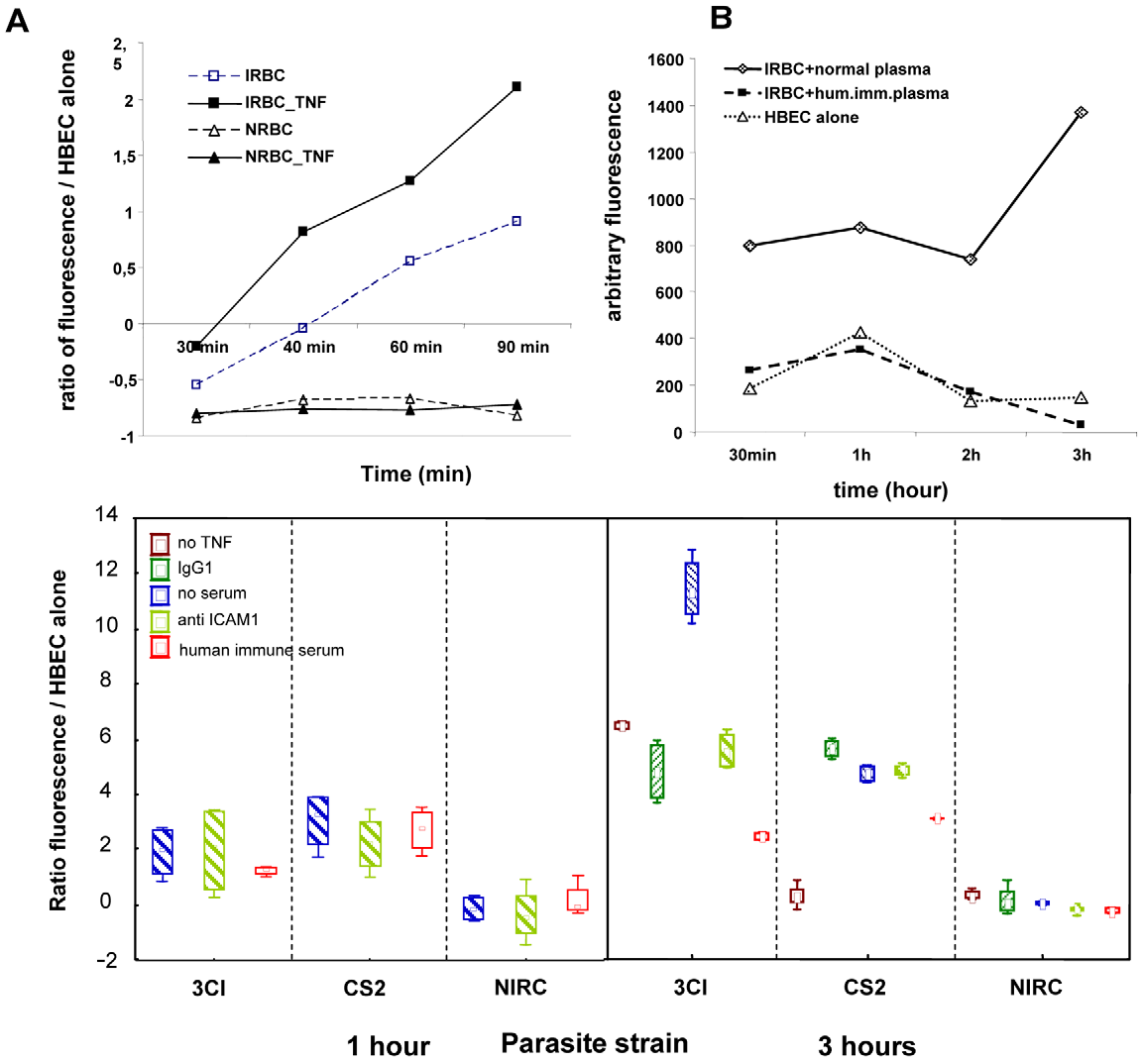
Quantification of the transfer of dye from PKH-labelled IRBC to HBECs during co-culture. HBECs D3 were grown to confluence in 24 wells plate, incubated overnight with 10ng/ml of TNF (except for control labelled “no TNF”) and co-cultured with PKH67-labelled IRBC for 30min to 3h before processing. Quantification of the fluorescence transferred from IRBC to HBECs was performed using an Optima Fluostar. Each figure summarizes four experiments (in triplicates). A) time dependent transfer of dye to the HBECs. IRBCs are incubated without (“IRBC”) or with (“IRBC-TNF”) previous activation of the cells with TNF, to highlight the effect of TFN on the increase of adhesion and transfer of dye; non infected red blood cells (NRBC) are incubated in the same conditions as control; fluorescence transfer was expressed as the ratio of fluorescence of the HBEC monolayer alone [(HBEC+IRBC−HBEC_alone)/HBEC_alone]. B) Effect of immune plasma on the time dependent transfer of dye to the HBECs. Control consists of HBECs incubated without IRBCs. It shows that pre-incubation of the IRBCs with immune (but not with non-immune) plasma inhibits adhesion of IRBC to the HBECs. C) shows effect of incubation of IRBCs with serums before co-culture with HBECs on the transfer of dye. “control” is incubation with “no-serum”. Immune plasma used had a strong effect (50%) on adhesion after 1 h or 3 h of co-culture. Anti-ICAM1 (5 µg/ml) had a clear but mild effect (25%) on adhesion of 3CI (selected to stick to ICAM1) but none on adhesion of CS2 (selected to stick to chondroitine sulphate). Anti-VCAM1 had rather no effect on the adhesion of the IRBC (not shown).

Upon incubation with human brain EC (HBEC 5i and hCMEC/D3 lines), IRBC bound tightly onto the endothelial surface. To study the transfer of IRBC constituents to EC we fluorescently-labelled both the IRBC membrane, using the membrane-intercalating agents PKH26 or PKH67, and the cytoplasm, using calcein-AM. We then followed IRBC material transfer by confocal microscopy and estimated the amount of fluorescence incorporated into the EC. As seen in Fig. 1, after 30 min, IRBC were seen attached to hCMEC/D3, with a halo-like diffusion from the IRBC PKH-labelled elements present on the HBEC surface around the area of attachment of IRBC (Fig. 1A–C). This process was enhanced after one hour of co-incubation, with a pattern of dense punctuate patches visible on the HBEC surface after 90 min (Fig. 1D)., After 3 h the fluorescent dye had migrated into the HBEC away from the original IRBC attachment point (Fig. 1E). The labelled elements transferred from IRBC were detected on the HBEC surface up to 24 h after their co-incubation, even if the IRBC had been removed from the culture at 3 h post-incubation. On the other hand, calcein remained localised within the IRBC for up to 1 h after incubation with HBEC (Fig. 1D). Calcein-labelled IRBC could still be detected as globular shapes on the HBEC, after 90 min of co-culture. However, after 3 h of incubation, the green dye was detectable in the HBEC far from the IRBC docking area (Fig. 1E). Time-dependent transfer of PKH67 dye to HBEC was quantified by the amount of fluorescence present in HBEC after extensive washing of the endothelial monolayer to remove any unbound IRBC (Fig. 2A). This transfer was significantly enhanced by TNF stimulation of the HBEC prior to incubation with IRBC (Fig. 2 A–B). No dye transfer was detected when HBEC were incubated either with fluorescent non-infected RBC (Fig. 2 A) or when the labelled IRBC were mildly trypsinised prior to incubation with HBEC (Data not shown, DNS).

**Figure 3:**
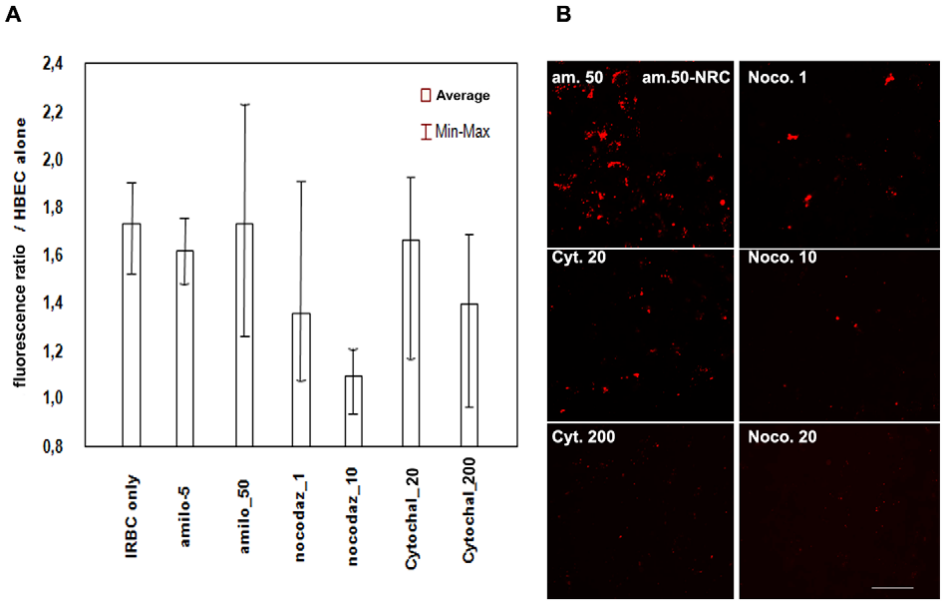
Effect of cytoskeleton inhibitors on the transfer of the membrane dye from IRBC to HBECs. Aspect of the HBEC-D3 monolayer after incubation with PHK67-labelled IRBCs (3Ci) for 3 h in the presence of various concentrations of inhibitors (nocodazole 1-10-20 µM; cytochalasine D 20–200 µM; Amiloride 5–50µM). Cultures were conducted in 24 well plates in standard conditions. A) Quantification of the fluorescence transferred from IRBC to the HBEC using an Optima Fluostar. For each concentration of inhibitors, fluorescence ratio was calculated as: (HBEC IRBC−HBEC alone)/HBEC alone); experiment was done 4 times in triplicates. B) Visualisation of the same HBEC monolayers prior to fluorescence quantification in Optima, with a Fluorescence Olympus IX71 (magnification 400) Bar: 100 µm.

We assessed cytoskeletal involvement in this transfer process by incubating the HBEC with optimal concentrations of cytoskeleton mobilisation inhibitors prior to co-culture with IRBC, followed by quantitation of the transferred fluorescence. Nocodazole (1 or 10 µM), a microtubule stabiliser, and cytochalasin D (20 or 200 µM), an actin reorganisation inhibitor, were the most effective in blocking the transfer of IRBC membrane elements, reaching a 6-fold inhibition (Fig. 3). Amiloride (5 or 50 µM), a micropinocytosis inhibitor, was less effective at blocking the transfer of IRBC membrane elements.

**Figure 4:**
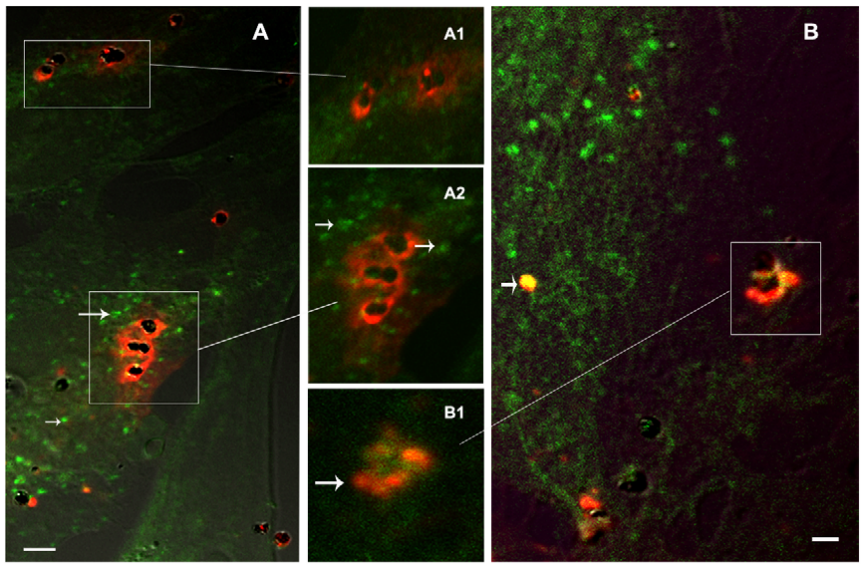
Transfer of material from IRBC membrane to HBEC early endosomes. After 1 or 3 h of co-culture, IRBC (3Ci) membrane components (red PKH26 labelling) are transferred to HBEC-D3 and partially to early endosomes (green anti-EEA1 labelling). HBEC-D3 monolayers are visualised after fixation, permeabilization, labelling and mounting as described. Hemozoin appears as black dots in the middle of IRBCs. Bar: 20 µm (A), 10 µM (B). A1 and B1-B2 are higher magnification of A and B, respectively. A) 1h incubation, trogocytosis like diffusion of PKH26 form IRBC on the HBECs membranes is still visible but no transfer of PKH26 was detected in the EE (arrow); B) after 3h of co-culture, PKH26 (red) from IRBC is partially transferred to EE (green) and merges as yellow labelling (arrow).

We attempted to identify the cellular compartment involved in the IRBC material transfer to the HBEC by selectively labelling early endosomes (EE), lysosomes or clathrin-coated pits. After less than 90 min of incubation, only the surface of HBEC appeared labelled with PKH26, in a trogocytosis-like process (Fig. 4 A). At longer incubation times (90 min to 3 h) PKH26 was partially detected in EE (Fig. 4 B) but not in either lysosomes or clathrin-coated vesicles (DNS).

### Adhesion of IRBC to HBEC is associated with transfer of malaria antigens

We evaluated the transfer of malaria antigens upon adhesion of IRBC to HBECs using a pool of human adult immune serum (HIS) originating from a dispensary in South Senegal, an area where malaria transmission is mesoendemic and where adults develop premunition against malaria. A pool of serum from age-matched adults from non malaria endemic areas was used as control. Western blot analyses indicated that the HIS pool recognised parasite antigens as well as parasite-encoded erythrocyte membrane proteins. In contrast, HIS could not detect any malaria antigens in proteins extracted from HBEC containing fluorescent compounds transferred from IRBC. This could be due either to sensitivity, with insufficient amounts of parasite proteins transferred to the HBEC to allow detection by Western blot or to degradation of the parasite antigens during the cell-to-cell transfer.

**Figure 5:**
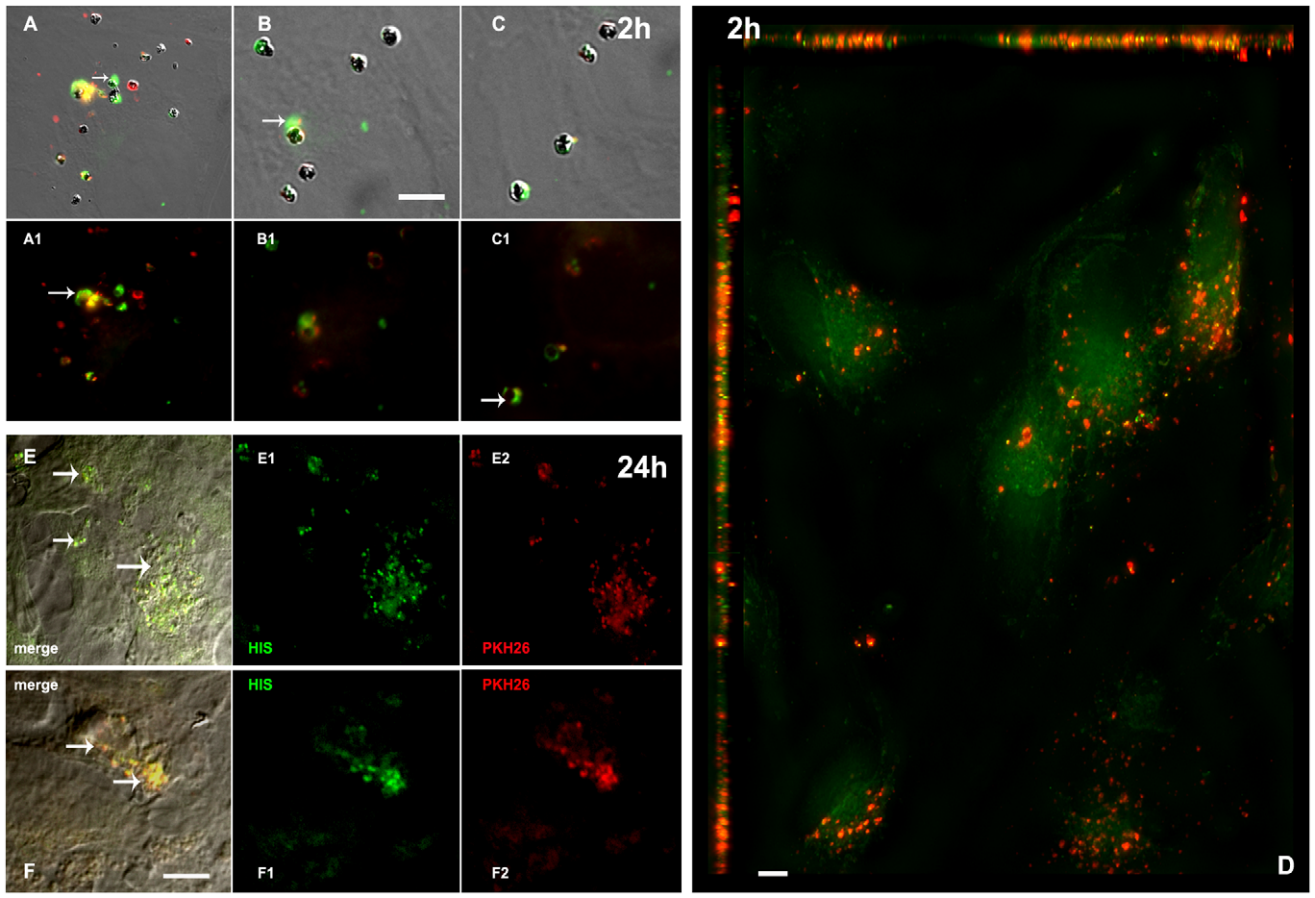
Transfer of malaria antigens to HBECs during co-culture with IRBCs. Figure shows HBEC surface with attached IRBC and diffusion of dye on the surface. PKH26(red)-labelled IRBCs (3Ci) were incubated with HBEC-D3 monolayers for 2h (A–D) and removed before an additional overnight culture of the cells (E–F). After fixation of IRBC and permeabilization of the cells, malaria antigens are detected using a pool of human immune serum from Senegalese patients (HIS),.. Remaining IRBCs appeared as yellow. HBEC monolayers are visualised with Olympus IX71 at magnification 1000 (A–C) or 600 (D–F). DIC and fluorescence are merged for A-B-C-E-F; only fluorescence is shown in A1-B1-C1, E1-2 and F1-2; Bar: 20µm (A–C), 50µM (D–F). A–C) early diffusion of malaria antigens (green and arrow) onto the surface of HBECs around the IRBC (arrow). PKH-labelled compounds and malaria antigens migrated mostly separately. IRBC themselves can be seen as yellow spots prior diffusion of compounds. D) 2D-reconstruction after deconvolution analysis of z-stacks. Nearest neighbour deconvolution analysis was applied onto 20 sections of 0.5 µm to generate 2D view. IRBC PKH-labelled (red) membrane lipids clearly diffused separately from antigenic compounds (green); upper and left bar show reconstitution of section of HBECs and highlight separate diffusion of red and green in the HBECs. A slight green labeling can be seen on the HBEC membrane may be due to other antigenic malarial components, possible including soluble components released from IRBC during incubation and covering HBEC surface. E–F) Persistence of malaria antigens and PKH-26: malaria antigens (green) and IRBC membrane compounds (red) are detected in the HBECs after an additional overnight incubation of the cells. (yellow merge colour) ). The labelling can only be seen after permeabilization of the HBEC which supports an intracellular localisation of the malaria antigens in vesicular structures (arrow).

However, using immunofluorescence deconvolution microscopy, HIS readily detected parasite proteins transferred to HBEC. It strongly detected the IRBC, but not the non-infected RBC on the HBEC surface (Fig. 5). This labelling can be detected even without any permeabilization of the cell membrane with triton. After 30 to 90 min of co-culture, HIS revealed a malarial antigen pattern on HBEC closely related to that obtained with PKH26-labelled IRBC (Fig. 5 A–C). However, after a 2 h co-culture, PKH-labelled elements and malarial antigens could clearly be located in different areas on the surface of HBEC (Fig. 5 A–C and Fig. 5F). This disparity may result from the different metabolic pathways to which the lipids and proteins transferred from the IRBC are subjected. At 24 h post-incubation, malarial antigens were still detected inside HBEC (Fig. 5 D–E). However there was no longer any detectable labelling of the HBEC plasma membrane as it is not detectable without permeabilization of the cell membrane with triton data not shown). We also used HIS to evaluate the role of IRBC-surface antigens in IRBC/HBEC adhesion with a view to determine the putative role of the antibodies present in immune sera in protection against CM. Pre-incubation of IRBC with HIS, but not with non-immune serum, abrogated IRBC adhesion on HBEC as well as the transfer of PKH26-labelled material, as observed by both microscopy (data not shown) and by fluorescence quantification (Fig. 2 B–C). As expected this pre-incubation had no effect on the adhesion of non-infected RBC used as control (Fig. 2C). This observation strongly supports a role for humoral immunity in the protection against the IRBC/HBEC binding process.

### IRBC/HBEC interaction during adhesion involves a transmigration-like structure

**Figure 6:**
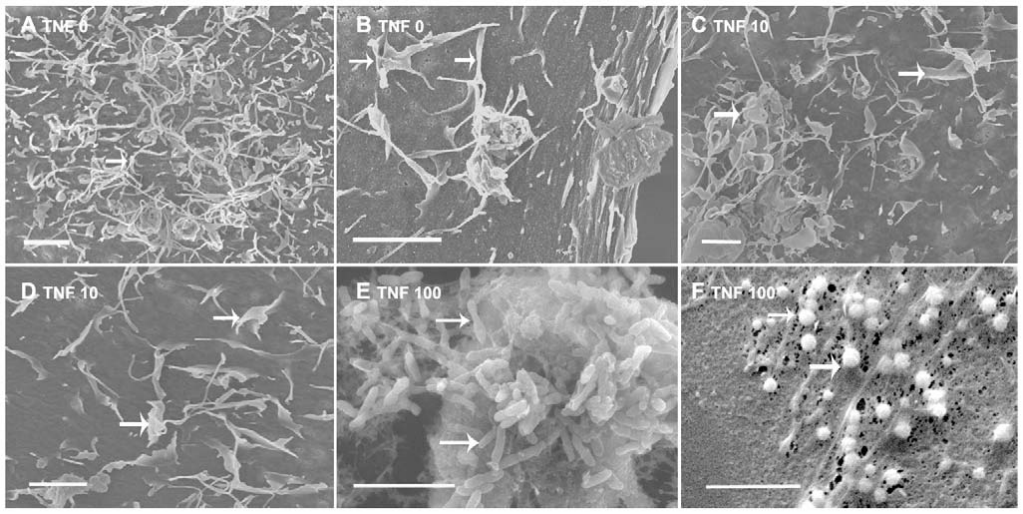
Scanning electron microscopy of HBECs monolayers. HBECs 5i were grown to confluence and incubated overnight with 10 ng/ml (C–D) or 100 ng/ml (E–F) TNF, or without TNF before processing (see M&M) and observed with a Philips XL30 at 10 kV. Bar: 2 µm. A–B) show digitations and podocytes (arrow) on the surface of HBECs, B–C) show leaf-like enlargement of the digitations (arrow), E–F) show enlargement of the digitations (arrow) and production of microparticles (arrow).

**Figure 7:**
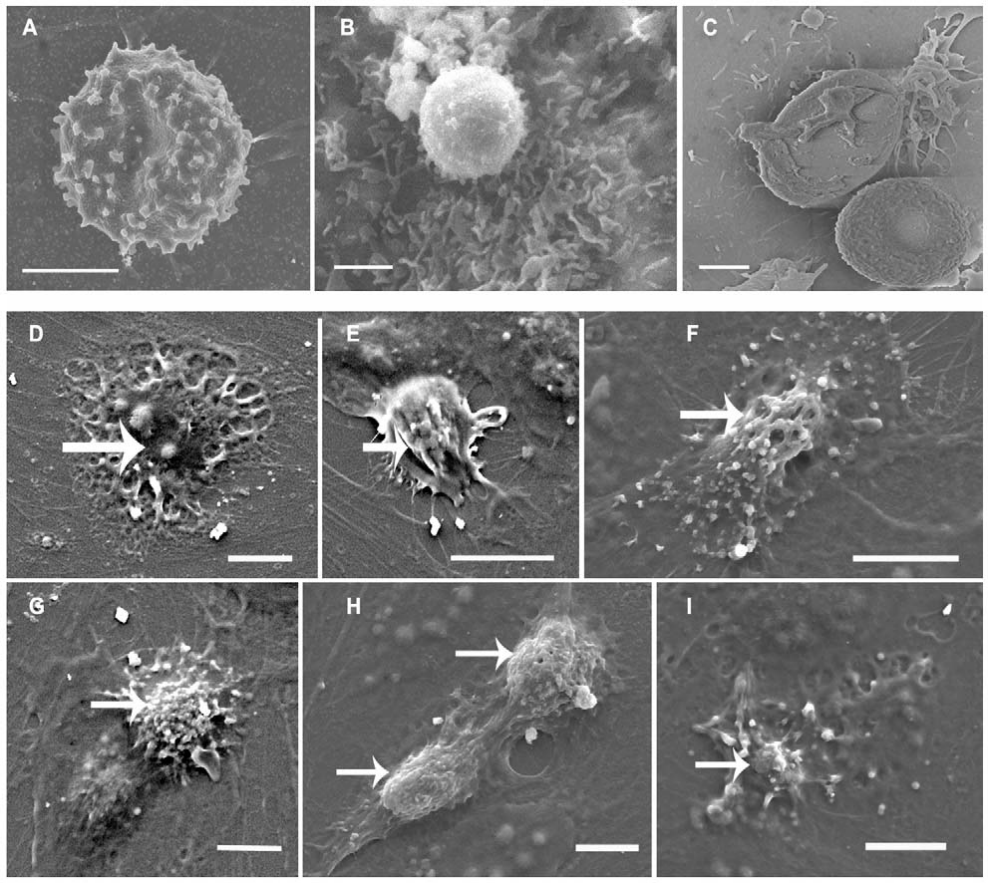
Scanning electromicroscopy of HBECs monolayers incubated with IRBC. HBECs-5i (B–C) and D3 (A, D–I) were grown to confluence on coverslides, incubated overnight with 10ng/ml of TNF and incubated with IRBC (3Ci) for 40 min (A–C) or 90 min (D–I) before processing and observation with a Philips XL30 at 10 kV. A–C) show aspects of the first step of adhesion of IRBCs onto HBECs with (B–C) or without (A) microvilli. D–I) show the engulfing process of IRBC with development of fillipods (D), formation of the cup (E) , and engulfing (F–I). (arrow, engulfing structure) Bar: 2 µm (A–B), 10 µm (D–I).

At present, the fine IRBC/EC contact structure had not been totally described. We conducted a detailed study of the IRBC/EC adhesion area using two HBEC cells lines, named 5i and hCMEC/D3 which presented marked differences in their surface morphology in standard culture conditions. HBEC-5i have defined surface structures, such as microvilli, podocytes and cups (Fig. 6), close to those described *in vivo* [14]–[15]. In the opposite way, as described by Weksler (2005), cultured hCMEC/D3 present a very smooth surface (Fig. 7A). Structures found on the 5i surface in resting condition could be related to a partial activation of these cells, as suggested by their high basal level of ICAM-1 expression. Overnight incubation of the HBEC 5i cells with 10 to 100 ng/ml of TNF induced dramatic changes in these structures, with an enlargement of the microvilli into leaf-like structures (at 10 ng/ml) (Fig. 6C–D) and to finger-like structures (at 100 ng/ml) (Fig. 6E). The highest TNF concentration induced large areas of bubbling microparticles, as described earlier [16]–[17] (Fig. 6F).

**Figure 8:**
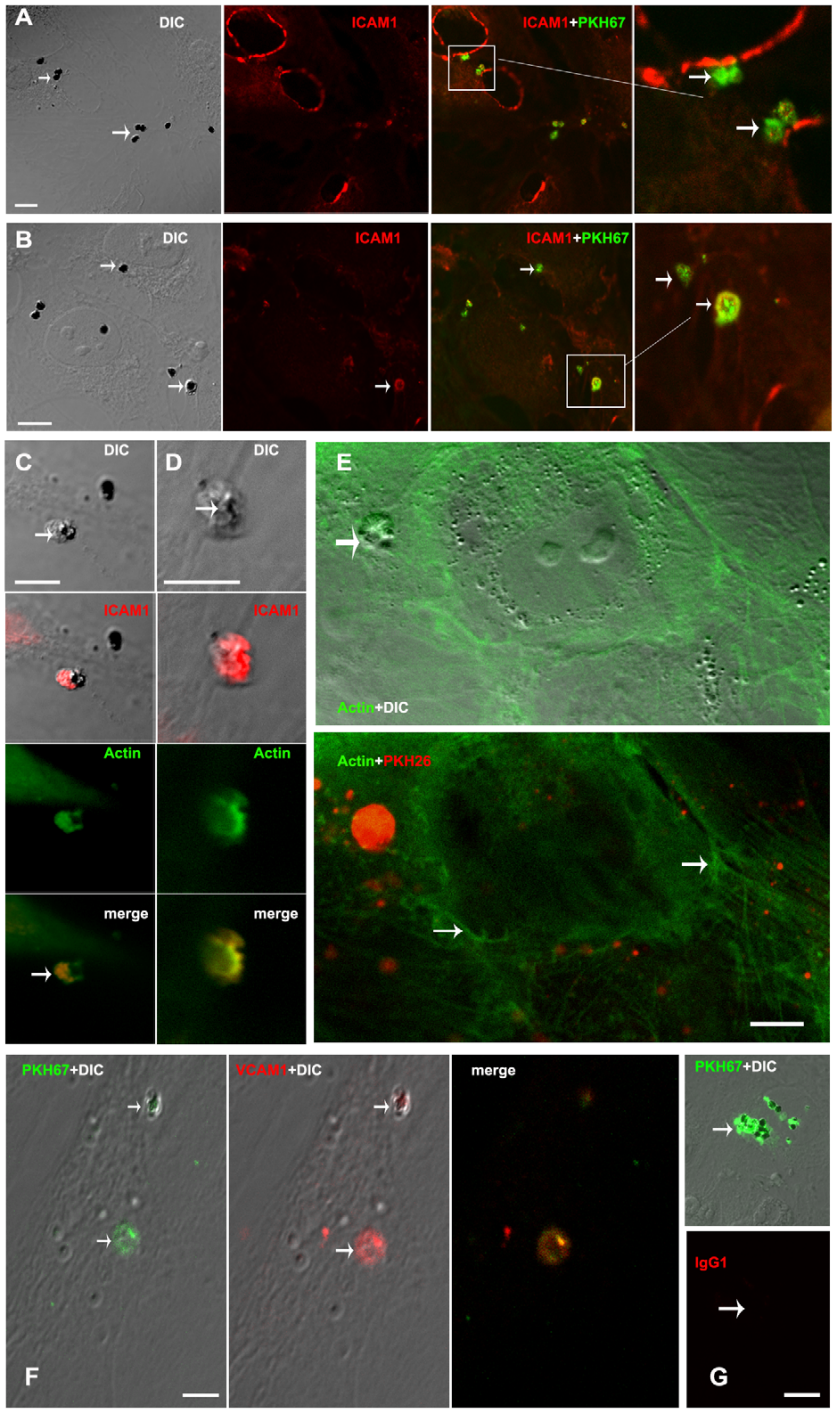
Involvement of ICAM-1, actin and VCAM-1 in the binding of IRBCs on HBECs. HBECs D3 were grown to confluence, incubated overnight with 10 ng/ml TNF and co-cultured with IRBCs for 90 min before processing and labelling with anti-ICAM-1, phalloidin or anti-VCAM-1.. Bar: 20µm (A, B, E–G), 10µm (C–D). A–B) show PKH-labelled IRBC stacked onto the HBECs and colocalized with ICAM-1 labelling. ICAM-1 was both detected on the surface of the HBECs and in the engulfing cup of the IRBCs, C–D) show engulfment of an IRBC by HBEC. ICAM-1 and actin are co-localized in the engulfing cup (arrow). E) shows stress actin fibres in HBECs (arrow) and accumulation of actin in the engulfing cup (arrow) of PKH-labelled IRBCs. F) same as A–B but with VCAM1 detection in the engulfing cup (arrow). G) same as A–B with control mouse IgG1 isotype labelling showing no signal.

We observed differences in the first contact between IRBC and HBEC according to the type of HBEC lines used and the type of surface involved. In the case of HBEC-5i, IRBC adhesion occurred on the microvilli, in a capture-like process (Fig. 7B–C). The subsequent engulfing structure appeared to be closely related to that described for leukocytes (Fig. 7D–I). The microvilli were progressively emitted from the surface at the same time as a transmigration cup-like structure was formed (Fig. 7D–E). This cup-like structure progressively covered and engulfed the IRBC (Fig. 7F–I). Fig. 8C–D shows a confocal image of IRBCs in these cup-like structures.

### ICAM-1 is involved in the binding of 3Ci IRBC

FRC 3Ci is a parasite strain selected for its ability to bind to CD36 and ICAM-1, whereas the CS2 strain mainly binds to CSA. hCMEC/D3 are known to poorly express CD36, a fact we confirmed by flow cytometry (DNS). However both hCMEC/D3 and -5i are known to express ICAM-1 on their plasma membrane, especially following stimulation by TNF [17]. We analysed whether actin, ICAM-1 and/or VCAM-1 play an active role in this engulfing process by imaging their distribution during the co-culture.

Actin displayed a sub-membranous labelling pattern (Fig. 8E), originally described as stress fibres [18]. However, a crown of actin was also detected, preferentially located around the parasite in the digitations of the cup (Fig. 8C–E). ICAM-1 displayed a pattern of membrane folds on HBEC or at the border of cells when there are not fully confluent (Fig. 8A–B). At higher magnification, was also detected at the bottom of the cup-like structures, under the IRBC and forming part of the cup digitations themselves (Fig. 8A–D). While VCAM-1 was weakly expressed on the HBEC surface, a definite labelling was found at the bottom but not on the borders of the of cups (Fig. 8F). In combination, these results suggest that IRBC engulfment is a process closely related to leukocyte transmigration, which involves adhesion to ICAM-1. On the IRBC side, membrane molecules such as PfEMP1 are likely to be involved in the binding to ICAM-1, and their removal by trypsin would explain the abolishment of the binding to HBEC.

Consistent with this, hCMEC/D3 incubated with anti-ICAM-1, but not with anti-VCAM-1, antibody prior to co-culture with IRBC displayed reduced binding of 3Ci, but not of CS2 IRBC (Fig. 2C). However, other adhesion molecules could be involved in this EC/IRBC binding which could explain this partial inhibition.

### Adhesion of IRBC induces opening of hCMEC/D3 intercellular junctions

**Figure 9:**
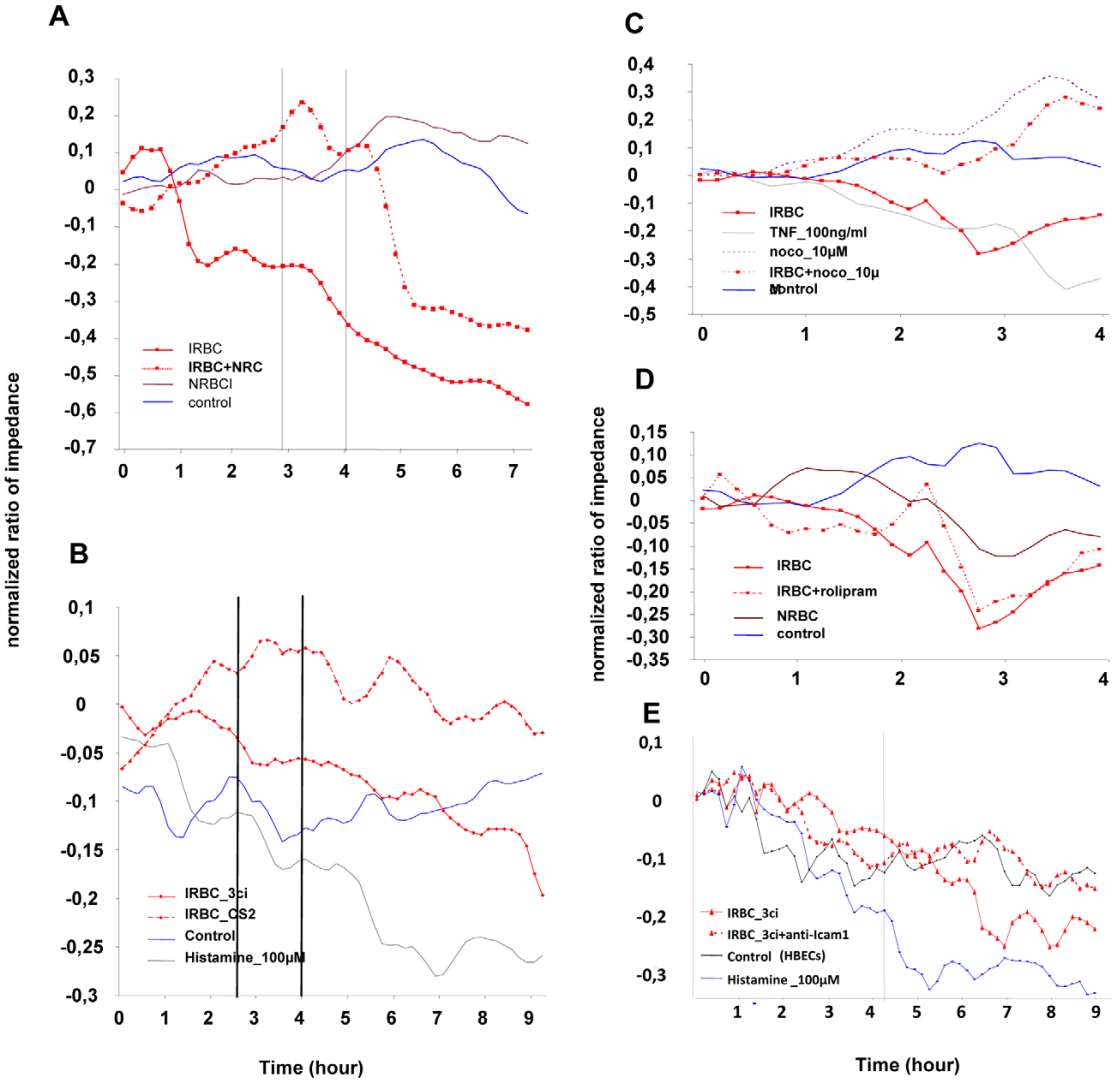
Measurement of the impedance of the HBEC monolayers during coculture with IRBC. Opening of the intercellular junctions was estimated by dynamic measurement of the impedance of the monolayer every 10 min for 24 h after the beginning of the co-culture on an ECIS instrument. HBEC monolayers are grown to confluence and co-cultured with IRBC (3Ci- or CS2) or NRBC. Red blood cells were removed from the HBECs by gentle wash, 4 hours after beginning of incubation. All the monolayers are incubated with 10 ng/ml of TNF prior commencement of the co-culture with no effect on the impedance (see Controls). Experiments were done more than 10 times but only illustrative curves are shown.. Nocodazole (10 µM), rolipram (10 µM) or anti-ICAM-1 (5 µg/ml) were added 30min prior beginning of the co-culture and let in the medium during all the time. TNF (100 ng/ml) and Histamine (100 µM) were used as positive control to induce opening of the junction. A) show decrease of impedance when HBEC monolayer is co-cultured with IRBC(3Ci) or IRBC(3Ci)+NRBC (vol/vol), but not when co-cultured with normal RBC). Impedance is related to parasiteamia as the effect is less when IRBC are diluted with NRBC; B) decrease of impedance when HBEC monolayer is co-cultured with IRBC(3Ci) but not with IRBC(CS2) which do not bind tightly to HBECs; C) inhibition of the IRBC(3Ci) effect on impedance by previous incubation of the monolayer with nocodazole; D) absence of inhibitory effect of rolipram on impedance in the same setting as C); E) absence of inhibitory effect of anti-ICAM1 on impedance in the same setting as C).

A major event driving brain pathophysiology during malaria infection is the opening of the blood-brain barrier (BBB). We used hCMEC/D3, capable of forming a monolayer and establishing tight intercellular junctions involving VE-cadherin and ZO-1 [19], to analyze the signals responsible for opening of intercellular junctions. Electric cell–substrate impedance sensing (ECIS) of the HBEC monolayer was used to assess trans-endothelial electrical resistance (TEER), which reflects opening of the intercellular junctions. Experiments were done more than ten times, but only illustrations are showed in figures as summarizing ECIS data is not accurate. Four days after seeding, impedance of the monolayer was stable, with only minor fluctuations (see “control” Fig. 9A–D). Overnight pre-activation of the confluent monolayer with 10 ng TNF/ml did not cause any modification in the TEER (DNS). Histamine (100 µM) was used as a positive control and induced a two-phase junction opening process, with a rapid decrease in TEER, within 30 min of addition, followed by a slow and steady TEER decrease, lasting for several hours (Fig. 9B).

We found that 3Ci-IRBC, but not non-infected RBC, induced a opening of the junctions after 2 h of incubation (Fig. 9A). This opening depended on parasiteamia and lasted for over 24 h, even if the RBCs were carefully removed after 4 h of incubation (Fig. 9A). In contrast, CS2 IRBC were only capable of inducing a minor decrease in the monolayer's TEER (Fig. 9B). When the RBCs were not removed after just 4 h but co-cultured with the HBEC monolayer overnight, they induced a slight decrease in TEER which was apparent even with non-infected RBC (DNS). This effect may be due to changes in the culture medium composition due to RBC lysis.

We then proceeded to use the ECIS to test compounds that would inhibit the junction-opening effect of the 3Ci IRBC. Pre-incubation of the HBEC monolayer with 10 µM nocodazole abrogated the IRBC-induced junction opening (Fig. 9C). In contrast, 10 µM rolipram, a compound known to strengthen junctions by maintaining/stimulating cAMP signalling, had no effect on 3Ci IRBC-induced opening of the junctions (Fig. 9D). Pre-incubation of HBEC with anti-ICAM-1 antibodies (10 µg/ml) had no effect on monolayer TEER (DNS), but partially inhibited the opening of the junctions induced by 3Ci IRBC (Fig. 9E). This effect is consistent with the partial inhibition that the anti-ICAM-1 antibodies caused on the IRBC binding to HBEC.

## Discussion

### A trogocytosis-like mechanism is the first step in IRBC-HBEC interaction

Here we demonstrate that the first step during the IRBC/HBEC interaction is a diffusion of IRBC membrane elements on the surface of HBEC, with features similar to those of trogocytosis. This process originally described during amoeba infection [20], is also used by all hemopoietic cells [21] and plays a major regulatory role in immunity.

Both T and B cells acquire their antigens by trogocytosis, in the same way that Natural Killer (NK) cells modulate IL-4-polarised monocytes [22], regulatory T cells acquire their allo-antigens to kill syngeneic CD8 T cells [23], and CTLs capture membrane fragments from their targets [24]. Additionally, T cells, NK, gamma-delta T cells and monocytes use trogocytosis to interact with cancer cells [12], [25]. However, it was not until this year that this mechanism was encountered in non-immune cells when Waschbish et al found that the capture of myoblast membrane patches by T-cells occurred by trogocytosis [26]. Here we describe, for the first time, this trogocytosis-like interaction process between two non-immune cells, i.e. endothelial cells and red blood cells. Using human malaria-immune serum, we were able to demonstrate diffusion of malaria antigens from IRBC to HBEC during the early stage of trogocytosis. This process could result, during malaria infection, in the transfer of malaria antigens to HBEC during short interactions, such as the rolling of infected cells on the endothelium. It has also been shown that in T cells [27] trogocytosis requires actin polymerization and involves kinase signaling pathways. Our results, showing that both nocodazole and cytochalasin-D were capable of inhibiting this process, as well as the actin redistribution observed by microscopy, strongly support actin involvement in the HBEC/IRBC interaction.

### IRBC-HBEC interaction results in the formation of a transmigration-like cup

After 1 to 3 h of co-culture, the contact between IRBC and HBEC became tighter and involved the formation of an engulfing cup-like structure. The formation of the cup was morphologically related to the structure formed during leukocyte transmigration. We found reorganisation of actin in the protrusions of the cup as well as a localized concentration of ICAM-1, and, to a lesser extent, of VCAM-1, in the bottom of the cup. This engulfing process has been analyzed in depth for leukocyte transmigration. During this process, a cup is formed with projections surrounding the leukocyte [9]–[10]. These projections were enriched in actin, but not microtubules, and required both intracellular calcium mobilisation and intact microfilament and microtubule cytoskeletons [28]. Disruption of these projections with cytochalasin D or colchicine had no affect on the adhesion of leukocytes but affected the cup formation itself [29]. Importantly a similar engulfing process has already been described for pathogens such as bacteria [30]–[31] and yeasts [32]. Bacteria were previously described to aggregate, before engulfing, in a specific structure called “invasome” which is highly enriched in actin, ICAM-1 and phosphotyrosine [30]. Formation of the invasome was found to be inhibited by cytochalasin D but not by nocodazole. Here we describe a potentially similar cup-like formation and engulfing process during IRBC/HBEC interaction. However, the fact that both cytochalasin-D and nocodazole inhibited the transfer of material from IRBC to EC suggested that, in our case, there are most likely different steps involved in the interaction.

The type of adhesion molecules involved in this interaction process depends on the type of cells interacting with the EC. For leukocytes, in response to LFA-1 engagement, the endothelium forms an ICAM-1-enriched cup-like structure that surrounds adherent leukocytes. Polymorphonuclear neutrophils use ICAM-1, but not VCAM-1, to move across activated EC monolayers [33]. On the other hand, the transmigration of THP-1 cells was reduced only when VCAM-1 or both ICAM-1 and VCAM-1 were blocked [34]. Similarly, a large panel of molecules is involved in the adhesion of IRBC on EC, including PECAM-1, CD36, chondroitin-sulphate A, ICAM-1, thrombospondin, αvβ3 E-selectin, P-selectin, and VCAM-1 [35]–[36]. The data we obtained for the two different parasite strains; 3Ci (which binds ICAM-1 and CD36) and CS2 (which mainly binds chondroitin-sulphate A), illustrates this diversity of these interactions. However, ICAM1 is a major adhesion molecule in HBEC largely increased during TNF stimulation, thus facilitating leukocytes migration through HBEC-D3 [33]. Additionally, upregulation of ICAM-1 has been described on brain microvessels in patients who died with CM [37]–[38] and correlates with adhesion of IRBC and the severity of attack in patients [39]. Taken together, our results provide new explanations for this increase in malaria pathology.

### Malarial antigens are transferred from IRBC to endothelial cells

After adhesion, IRBC were progressively engulfed in the EC monolayer and subsequently altered. As revealed by the use of human immune sera, our results highlight the diffusion of malarial antigens into HBEC. These antigens were first transferred onto the endothelial surface, in a trogocytosis-like process. Later on, the membrane dye, the cytosolic material of the red blood cells and malaria antigens were recycled in the endosomal compartment of the HBEC. Of note they were readily detected in these cells up to 24 h after incubation.

As HBEC are known to be antigen-presenting cells [40], we attempted to detect the malaria antigens on their surface after a 24 h co-incubation. After this time, the anti-malaria serum pool very slightly detects malaria antigens on the HBEC surface, however this could be due to degradation of the IRBC proteins (and epitopes) by EC. The observed transfer of antigens to the EC involves dramatic implications for the interaction of EC with the immune system, as it could transform the EC into a new target for the immune response, especially during the rolling of immune cells on EC, and trigger major pathophysiological changes during CM. More experiments are required using immune cells from donors from endemic areas, to study the interaction between the immune system and the “IRBC-loaded” HBEC.

### Opening of HBEC intercellular junctions shortly follows IRBC adhesion and requires calcium mobilisation

As occurs with sepsis and viral infections, a major element in the pathophysiology of CM is the opening of the intercellular junctions. We report here that the opening of these junctions occurred shortly after the beginning of the IRBC/EC co-culture and was closely related to adhesion of the IRBC on the HBEC, as the non-infected RBC or non-binding IRBC, such as CS2, did not induce any significant junction opening. This also implies that neither metabolic modifications of the culture medium nor proteins secreted by the parasite were, in our culture conditions, sufficient to induce junction opening. A decrease in trans-endothelial electrical resistance (TEER) in the endothelial monolayer is well known during leukocyte transendothelial migration [41] and has also been suggested during *in vivo* infection of mice with *P. berghei* [42]–[44]. Three primary signaling pathways are activated by leukocyte adhesion: Rho GTPases, reactive oxygen species, and tyrosine phosphorylation of junctional proteins [45]. The pathways activated during both cup formation and opening of the EC junctions, need to be further explored for IRBC attachment and transmigration.

ICAM-1 engagement by leukocytes has been shown to enhance trans-endothelial permeability by tyrosine phosphorylation of VE-cadherin [46]. However, in our results, treatment with anti-ICAM-1 antibodies had no effect on the increase of permeability induced by leukocytes on HBEC-D3, although it decreased leukocyte migration [33]. Interaction of ICAM-1 and VCAM-1 with adhesive molecules regulates the different steps of diapedesis by modulating either i) the GTPase pathway (Rho and Rac) [29], [34] ii) and the MAP-kinase pathway (Ca++, CaMKK, and AMPK) [47]. In our study, the opening of the EC junctions was independent from cAMP activation and suggested a MAP kinase pathway activation, as previously reported for *P. falciparum* [48]. However, the direct pathogenic effect of IRBC adhesion on the HBEC TEER must also be taken into account, as it up-regulates several TNF-superfamily genes and apoptosis-related genes such as Bad, Bax, caspase-3, SARP2, DFF45/ICAD, IFN-receptor2, Bcl-w, Bik, and iNOS [49]. This could account for the increase of permeability of the HBEC monolayer we observed after 6 or 8 h of IRBC/HBEC co-culture.

### What relevance during cerebral malaria?

Adhesion of IRBC on EC is a key step in the life cycle of the plasmodium parasite and this study highlights new implications for this adhesion. We showed that a first step of rapid transfer of material from IRBC to HBEC presented features of a trogocytosis-like mechanism. This was shortly followed by a tighter adhesion, which appears to divert the natural transmigration pathway of leukocyte-EC and involves a cup-like engulfing process. Malarial antigens then entered the HBEC endosomal pathways and were detected inside the HBEC up to 24 h later. This could be followed by their presentation to the immune system. IRBC transfer was closely followed by a rapid opening of the EC intercellular junctions, an event that may contribute to cerebral oedema. All the mechanisms hereby described can have dramatic implications in the pathophysiology of CM.

The relevance of these in vitro observations during CM is first supported by the experimental conditions used. Activation of the ECs and detection of TNF secreting monocytes in brains vessels in the same time as IRBC were reported by Pongponratn and Porta et al [37], [50] who showed images of IRBC sequestered in vessels in the same time as leukocytes. Sequestration of IRBC and CM seem closely related to TNF overproduction, as reported by Grau et al and Kwiatkowski et al [51], [52].

The range of TNF we used to activate ECs and inducing adhesion of IRBC is the same as already detected in vivo (100 pg/ml with high range near 500 pg/ml [53], [54]). This elevated level was also detected in Cerebrospinal Fluid [55], [56] especially in patients who died from CM. Upregulation of TNF-receptor 2 (TNFR2) seems also related to CM [57].

Along the same line, focal induction of ICAM-1 expression in infected brain vessels was also reported years ago. Brown et al [58] described activation of EC and macrophages and disruption of endothelial intercellular junctions in vessels containing sequestered parasitized erythrocytes. These findings suggest that BBB breakdown occurs in areas of parasite sequestration during CM in African children. Porta et al [37], showed CD68 leukocytes coexisting with infected erythrocytes in capillaries, whereas in venules the monocyte population outnumbered the erythrocytes. They also showed expression of ICAM-1 on EC surface in vessels with sequestered cells but not in unaffected vessels. Similarly, Esslinger et al reported that in vivo stimulation of human vascular EC with *P. falciparum*-infected erythrocytes resulted in the non-transient up-regulation of ICAM-1 expression on endothelial surfaces [59]. The soluble form of ICAM-1was also found significantly higher during acute malaria in children and correlated with levels of TNF, IL-1 alpha and interferon gamma [60]. Clark et al [61] showed inducible nitric oxide synthase staining of ECs which suggested intense inflammatory mediator activity. These alterations can be detected in other organs than brain especially in children who died during severe malaria without true CM.

Deposits of malaria antigens in vessels were also reported earlier during autopsies of patients dying from CM. Pongponratn et al first reported images of IRBC sequestered in vessels some associated with or beneath ECs [50]. Boonpucknavig et al demonstrated intense deposition of *P. falciparum* antigens, IgG and fibrin in cerebral vessels associated with hemorrhages [62]. Immunofluorescent studies also demonstrated the extravascular deposits of *P. falciparum* granular antigens associated with acute inflammatory lesions in cerebral tissue. IgE was also reported in these depositions especially in the white matter [63]–[65]. They were also found beneath ECs suggesting transfer of material. In the same line pLDH or pAldolase were detected in a variety of organs during CM but were most abundant in the blood vessels of brain, heart, and lung tissues, also detectable in ECs [66].

All these data strongly suggest transfer of malaria antigen in or beneath the EC wall in the brain. Our *in vitro* observations are thus relevant in regard to these *in vivo* findings and can explain a part of the pathophysiology of CM. Presentation of malaria antigens by ECs to immune cells and activation of cytotoxic mechanisms could be another step in the explanation of this pathology. Analysis of this mechanism will require malaria immune cells from tropical area countries and is currently in process.

## Materials and Methods

### Materials

The following antibodies and dyes used were: PKH-26 or -67 (MINI26-1KT or 67-IKT) and Phalloidin-FITC (P5282) from Sigma; anti-ICAM1 (0544) and anti-VCAM1 (1244) from Immunotech; ER tracker red BodiPyTR (E34250), Calcein-AM (C3099) and Lysotracker Red DND-99 (L7528) from Invitrogen; anti-EEA1 (610457) and anti-clathrin (07339) from BD Bioscience; Hoechst 33258; anti-human-IgG FITC (733175) and anti-glycophorin A (PN IM2210) from Beckman Coulter. Other compounds used were: albuMAX II (10021-037 Gibco), HMDS hexamethyldisilazane (H4875, Sigma); collagen (BD Biosciences); TNF (PreproTech, 300-01A).

### HBEC and IRBC cell culture

Immortalised human brain endothelial cells 5i (CDC Atlanta) were grown in DMEM/F12, whereas hCMEC/D3 [19] were grown in EBM-2 medium (Lonza CC-3156), in 24 well plates or on glass cover-slips coated with collagen. TNF activation of HBEC was carried out by treating the cells with 10ng/ml TNF for 18h. Plasmodium falciparum strains FCR 3Ci and CS2 (kindly donated by S. Rogerson) were grown in RPMI+0.5% Albumax, as previously described [67] and periodically selected for knob expression [68] Late stage IRCB were selected and concentrated using Automacs (MiltenyR), according to manufacturer instructions, to an average of 80–90% parasitaemia. The IRBC were then co-incubated with HBEC [69] at a 20 RBC: 1 HBEC ratio (i.e. 2×10^6^ IRBC per well in a 24-well plate). For overnight incubations, non-adherent RBCs were gently removed from the HBEC, after 4 hours of incubation, by washing the cells three times with pre-warmed medium.

### Microscopy studies

For trogocytosis studies, IRBC/RBC were labelled, according to manufacturers' instructions, with either PKH-26 or -67 [70], calcein-AM or Hoechst. Labelled RBCs were incubated, at 37°C for 30min, in parasite media prior to the last wash. Immunofluorescence detection was carried out on cells fixed in 2% paraformaldehyde (PAF) for 10min. Cells were treated with 25mM NH_4_Cl, permeabilized in 0.1% Triton X-100, and incubated for 30min in 3% bovine serum albumin (BSA) prior to antibody reactions (see list below) for 45min (in PBS containing 0.3% BSA). Detection of *P. falciparum* (Pf) antigens was performed using a pool of 15 human plasma samples selected out of 100 samples from Senegalese Pf-immune adults (used at 1∶500). The samples were selected for their high titer (>1024) in plasmodium antibodies [71] and for their low background on HBEC (checked individually by immunofluorescence). A pool of plasma from non-immune adults was used as a negative control. For microscopy examination cover-slips were mounted in moviol and examined either under an Olympus FV1000 confocal microscope (magnification 600 or 1000) or on an Olympus-IX71 fluorescent microscope equipped with a F-View CCD camera (Soft Imaging Sys.). Laser 405nm was used with Differential Interferential Contrast (DIC) to generate bright field images. Quantitation of fluorescence was carried out in 24-wells plates on a Fluostar Optimax Spectrophotometre (BMG LabTech) at the relevant wavelengths (100 spots/well were read).

For scanning electron-microscopy, HBEC were grown on 10 mm glass coverslides in 24-well plates for 5 days. They are co-cultured with IRBC as previously described. were fixed first in PAF 2% for 10 min and glutaraldehyde 2% in cacodylate buffer for 30min, followed by potassium ferrocyanate–osmium (1% each) post-fixation. Dehydration was performed in grading alcohols, with a final step in HMDS for 3 min.

### Transendothelial resistance measurement

Transendothelial impedance was measured every 10 minutes over the course of experiments using electric cell–substrate impedance sensing (ECIS) system. hCMEC/D3 were seeded at 20,000 cells/wells in 8-well slides and allowed to grow for 3 to 4 days, until confluent, in complete HBEC medium. Confluent hCMEC/D3 monolayers (estimated 100,000 cells/well) were activated with TNF (10 ng/ml) for 18 hours. Drugs were then added directly to wells 40 min prior to addition of IRBC (3Ci- or CS2) or NRBC at a ratio of 20 IRBC/cell. Red blood cells were removed from the HBECs by gentle washing, 4 hours after beginning of incubation. Histamine was used as a positive control for TEER modification. For each well, impedance at time t was normalized according to the impedance of the well at the beginning of the co-culture (t0) and plotted according to time as: (I(t)−I(t0))/I(t0). I(0) was evaluated as the mean impedance over 50 min just before beginning of experiments.
